# Evaluating of IAQ-Index and TVOC Parameter-Based Sensors for Hazardous Gases Detection and Alarming Systems

**DOI:** 10.3390/s22041473

**Published:** 2022-02-14

**Authors:** Mohammed Faeik Ruzaij Al-Okby, Sebastian Neubert, Thomas Roddelkopf, Heidi Fleischer, Kerstin Thurow

**Affiliations:** 1 Technical Institute of Babylon, Al-Furat Al-Awsat Technical University (ATU), Kufa 54003, Iraq; 2 Center for Life Science Automation (Celisca), University of Rostock, 18119 Rostock, Germany; kerstin.thurow@celisca.de; 3 Leibniz Institute for Baltic Sea Research Warnemünde, 18119 Rostock, Germany; sebastian.neubert@io-warnemuende.de; 4 Institute of Automation, University of Rostock, 18119 Rostock, Germany; thomas.roddelkopf@celisca.de (T.R.); heidi.fleischer@uni-rostock.de (H.F.)

**Keywords:** gas sensors, hazardous gases, alarming system, indoor air quality index (IAQ-index), internet of things (IoT), total volatile organic materials (TVOCs)

## Abstract

The measurement of air quality parameters for indoor environments is of increasing importance to provide sufficient safety conditions for workers, especially in places including dangerous chemicals and materials such as laboratories, factories, and industrial locations. Indoor air quality index (IAQ-index) and total volatile organic Compounds (TVOC) are two important parameters to measure air impurities or air pollution. Both parameters are widely used in gases sensing applications. In this paper, the IAQ-index and TVOCs have been investigated to identify the best and most flexible solution for air quality threshold selection of hazardous/toxic gases detection and alarming systems. The TVOCs from the SGP30 gas sensor and the IAQ-index from the SGP40 gas sensor were tested with 12 different organic solvents. The two gas sensors are combined with an IoT-based microcontroller for data acquisition and data transfer to an IoT-cloud for further processing, storing, and monitoring purposes. Extensive tests of both sensors were carried out to determine the minimum detectable volume depending on the distance between the sensor node and the leakage source. The test scenarios included static tests in a classical chemical hood, as well as tests with a mobile robot in an automated sample preparation laboratory with different positions.

## 1. Introduction

The measurement of the indoor air quality parameters is one of the important factors for improvements of health condition and life quality in our world. Many commercial electronic products include sensors for estimating values for the indoor air quality such as IAQ-index/VOC index, TVOCs, equivalent CO_2_, equivalent Ethanol, equivalent Hydrogen, and others. In addition to the monitoring of air contamination in living environments, the measurements of the indoor air quality can be used effectively in occupational safety applications, especially in chemical laboratories, factories, and any locations that may use or store dangerous chemicals that can produce toxic/hazardous gases, and chemical vapors. The IAQ-index can be used as a reference or a threshold for triggering an alarm in case of any abnormal levels of air pollution. The early detection and alarming of toxic and hazardous gases can avoid dangerous situations with negative impact on workers and the environment.

Several methods, communication modules, sensor types, and hosted environments and processors have been used to build hazardous gases detection and alarming systems. Neubert et al. [1], proposed a flexible IoT-based sensor node for hazardous gas detection and monitoring. The system consists of two MOX (Metal Oxide Semiconductor) gas sensors which are BME688 (Bosch Sensortec, Reutlingen, Germany) and SGP30 (Sensirion AG, Stafa, Switzerland) combined with NXP ARM microcontroller. The system was tested with several VOCs as a standalone unit as well as hosted by a stationary and mobile robot. The acquired data were sent to an IoT-cloud for data monitoring and storing using a WROOM (Espressif Systems, Shanghai, China) Wi-Fi module.

Palacín et al. [2], proposed an application of MOX gas sensor array hosted by a robotic system for early gas leakage detection. In this case, 16 gas sensors from four types TGS 2600, 2602, 2611, 2620 (Figaro Engineering Inc., Osaka, Japan) have been assembled at one portable unit. The system has been tested with ethanol, and acetone. The system has been controlled using the ARM-based STM32F407VGT6 microcontroller. APR-02 personal robot was used for gas detection unit hosting and providing the power supply and communication with the control station.

Burgués et al. [3], presented a quadcopter-based hazardous gas localization and mapping system. The TGS 8100 (Figaro Engineering Inc., Osaka, Japan) MOX gas sensor was used and tested with ethanol. The system is controlled by a 32-bit STM32F405 ARM microcontroller (STMicroelectronics, Geneva, Switzerland). The acquired data can be transferred to the ground control station using a 2.4 GHz RF communication module.

Optical sensors technologies were used by several researchers for hazardous gases detection. This type of gas sensor is less influenced by temperature and humidity changes in the tested environments, and safer in case of flammable gases detection. Esfahani et al. [4], developed a tunable optical electronic nose for hazardous gases and vapors detection. The sensing element consists of four tunable non-dispersive infrared (NDIR) optical sensors with 3.1 to 10.5 µm effective wavelengths. Each optical sensor has an emitter-detector pair, which is encapsulated with the others’ sensors pair in one heated chamber. The system was tested with several gases and VOCs such as carbon dioxide, methane, ethanol, isopropanol, acetone, but also cola, orange juice, and coffee. The test results revealed that the system was able to detect and distinguish between the odors of the tested chemicals.

Shi et al. [5], proposed a low-cost, and simple design fiber-optic gas sensor for the detection of volatile organic compounds. The sensor is based on a polydimethylsiloxane (PDMS) self-assembled Fabry-Perot interferometer. The tested VOCs were injected into the testing gas chambers and a sensor probe was used to take the gas sample and send it to a spectrometer with a 0.02 nm wavelength. The sensor was tested with several VOCs such as ethanol, isopropanol, toluene, and ethyl acetate. The tests results show good sensor performance, high sensitivity, good stability, and rapid response.

Arroyo et al. [6], presented an air quality measurement system based on electrochemical and optical particle matter (PM) gas sensors. The system is a portable device that can provide various values for gas and environmental measurements such as the concentration of different gases/chemicals vapors, PM_2.5_, PM_10_, temperature, humidity, and location data. A 32-bit STM32L476 ultra-low-power microcontroller (STMicroelectronics, Plan-les-Ouates, Switzerland) was used for data processing and system management. The system was connected to an IoT cloud platform using GSM and Ethernet modules, and all acquired real-time data can be transferred directly to the IoT cloud via the internet. Two system prototypes were tested with several chemicals such as NO_2_, NO, CO, O_3_, PM_2.5_, and PM_10_, and the results show good performance.

In this paper, we investigate two MOX gas sensors SPG30, and SPG40 (Sensirion AG, Stäfa, Switzerland) for measuring the indoor air quality parameters, which are the IAQ-index and the total volatile organic compound TVOC. The sensors were tested with different VOCs materials in two different test environments. Various sensor positions on a fixed stand and mobile robot were used to compare the performance of the two sensors based on the measured parameters (IAQ-index and TVOC). The main goal of the proposed work is to evaluate, which parameter should be used in future design for hazardous gases detection and alarming systems. A combination of two different gas measurement parameters such as the IAQ-index and TVOC will allow the system to minimize false-positive as well as false-negative gas detection errors [7,8,9,10,11,12,13].

## 2. Materials and Methods

**Figure 1:**
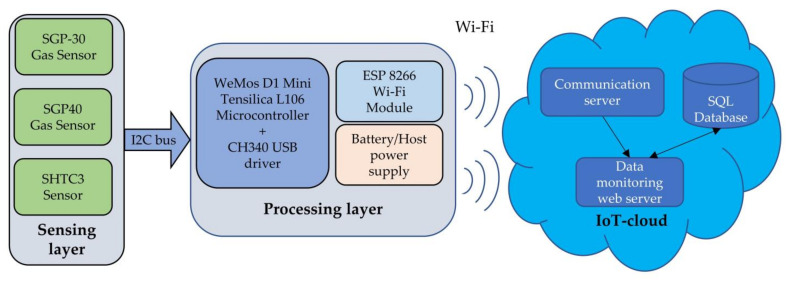
System structure.

The presented sensor module consists of three main layers. The sensing layer is responsible for sensing different parameters. This layer includes three sensors SGP30, SGP40, and SHTC3 (Sensirion AG, Stäfa, Switzerland) to measure IAQ-index, TVOC, and ambient environmental parameters (temperature, humidity), respectively. The second layer is the processing layer, which is responsible for receiving and processing the measured data from the sensing layer. The processing layer prepares the required gas and environmental measurements and send via a Wi-Fi module to the IoT cloud (third layer). It is a server system responsible for transfer, processing, storing, and provision of the data. A communication server on the third layer handles the communication to the second layer using UDP and HTTP POST protocol. The IoT-cloud stores the received and processed data in a database using an MS-SQL database server. The data can be accessed by the users from any location using a web server. Figure 1 explains the system structures. A detailed description of the systems modules is given in the following subsections.

### 2.1. SGP30 Gas Sensor

**Figure 2:**
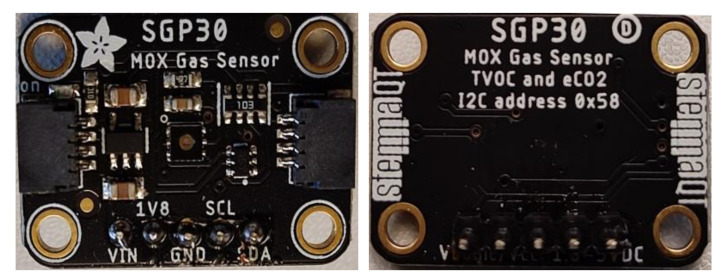
The used Adafruit SGP30 module board.

The SGP30 is a metal oxide semiconductor (MOX) gas sensor that is used for the measurement of air quality TVOC and equivalent CO_2_ (eCO_2_) parameters. It is a complete system on chip (SoC) that can easily be adapted with hosted devices using I^2^C communication protocol with I^2^C address (0x58). The sensor has a compact size design with 2.45 × 2.45 × 0.9 mm^3^, and has a low-power consumption (48 mA at 1.8 V) which makes it suitable for wearable and battery-powered based application and systems. The sensor can operate with a 1 Hz sample rate for both TVOC and eCO_2_ with an output range of 0–60 ppm for TVOC and 400–1000 for eCO_2_. In the proposed system, the Adafruit SGP30 module has been used (see Figure 2) [14].

### 2.2. SGP40 Gas Sensor

**Figure 3:**
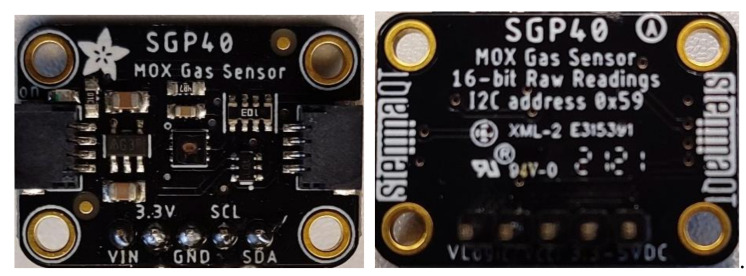
The used Adafruit SGP40 module board.

The SGP40 is a metal oxide semiconductor (MOX) gas sensor used for indoor air quality index IAQ-index (also called VOC index) measurements. It is a small (2.44 × 2.44 × 0.85 mm^3^) system on chip sensor, including a 6-pin DFN package. The sensor has a low-power consumption (2.6 mA at 3.3 V) and can be easily adapted with any hosting system using an I^2^C bus with (0x59) I2C address. The sensor sample rate for IAQ-Index is 1 Hz and the IAQ-Index ranges from 0–500. The compact size, lightweight, and low-power consumption make the sensor very suitable for battery power-based and wearable applications. The Adafruit SGP40 module board is used in the proposed system (see Figure 3) [15].

### 2.3. SHTC3 Environmental Sensor

**Figure 4:**
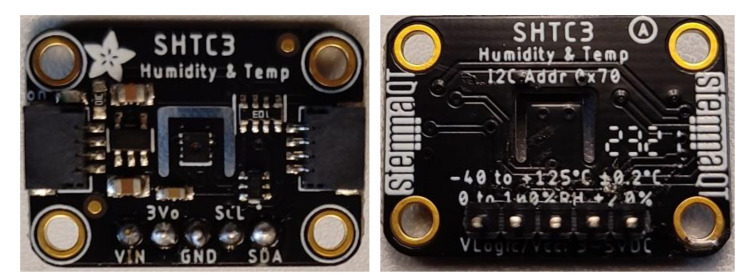
The used Adafruit SHTC3 module board.

The SHTC3 is a fully calibrated digital environmental sensor used for temperature (T) and relative humidity (RH) measurement. It is used for SGP30 and SGP40 calibration algorithms to reduce the measurement errors that can result from changes in the ambient humidity and temperature. It consists of a band gap temperature sensor, a capacitive humidity sensor, an A/D converter, and memory for calibration data. The sensor communicates with the hosted system using an I^2^C communication bus. The sensors are manufactured in a small DFN package of dimensions 2 × 2 × 0.75 mm^3^, and it is classified as ultra-low-power consumption sensor. The sensor can be powered by any source with a voltage range between 1.62 and 3.6 V). The operation measurement ranges are (1–100% RH) for relative humidity and (−40 to 125 °C). The sensor response time is 1 ms with an accuracy range of ±2% RH and ±0.2 °C for humidity and temperature, respectively. In the proposed system, the Adafruit SHTC3 module board has been used (see Figure 4) [16].

### 2.4. WeMos D1 Mini Development Board

**Figure 5:**
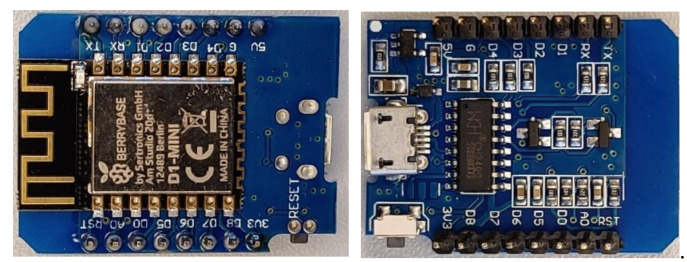
The used WeMos D1 Mini Development board.

The WeMos D1 Mini is a Wi-Fi-based microcontroller for IoT applications, including the chip ESP8266 (Espressif Systems, Shanghai, China). The ESP8266 is driven by the 32-bit Tensilica L106 processor (Tensilica Inc., San Jose, CA, USA) with a built-in TCP/IP networking software for a simple Wi-Fi connection. It has a 4 MB memory and can run with up to 160 MHz max speed. The WeMos D1 Mini provides all the required communication protocols, which are mainly the I^2^C bus to communicate with the sensing layer, the Wi-Fi protocol to communicate with the IoT-cloud, and the CH340 driver integrated circuit for establishing a simple communication between the WeMos board and computers. In addition to all the previous features, the WeMos D1 Mini provides a low cost (≈€4), small size (2.5 × 3 cm), and low-power consumption that make it a good choice for mobile devices, wearable electronics, and IoT applications. Figure 5 shows the used WeMos D1 Mini Development board [17].

## 3. Work Description

This work aims to evaluate two parameters used widely to measure air pollution/air impurity: indoor air quality index (IAQ-Index) and the total volatile organic compounds concentration (TVOC). The result of this study will help to choose the best parameter that can be used in hazardous gases detection and alarming system. The selected parameter will be used as thresholds for triggering alarms to alert people from hazardous and toxic gas leakages in a work environment. The IAQ-Index refers to the air quality/air pollution within and around the work areas. The IAQ-index does not have a measurement unit. The IAQ-Index range is bounded between 0–500 steps from the best to the worst air quality level (see Table 1 for IAQ-Index values, and it is categories) [18].

The second selected parameter is the TVOC. The TVOC refers to the concentration of volatile organic compounds, which are a group of organic chemicals that vaporize easily at room temperature. The abbreviation VOC is used for a large group of chemicals such as ethanol, acetone, hexane, benzene, etc. The abbreviation TVOC refers to the presence of several VOCs in the air sample. TVOC can be measured in milligrams per cubic meter (mg/m^3^) or in parts per million (ppm) which we used in the presented work. Table 1 explains an approximation of TVOC levels compared to the IAQ-Index levels [19,20,21,22,23].

**Figure 6:**
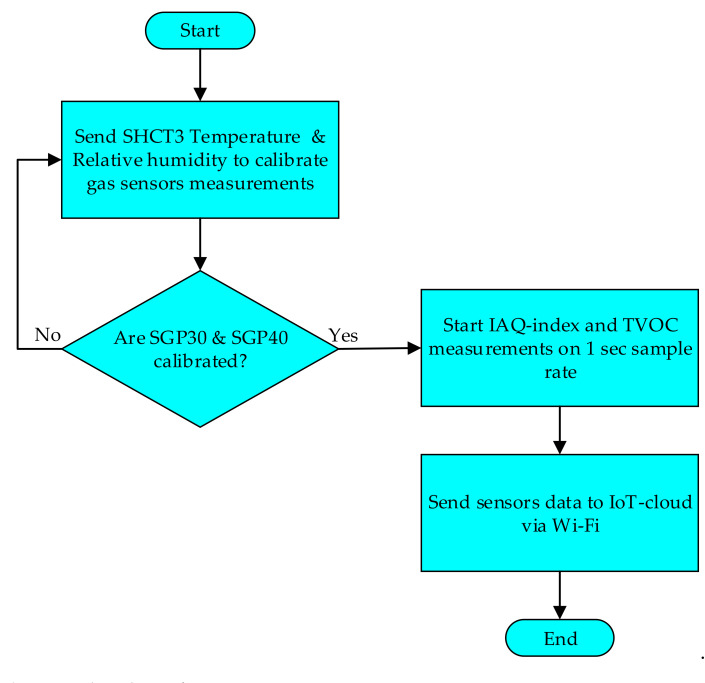
Flowchart of measurements process.

The SGP30 and SGP40 gas sensors measure the TVOC and IAQ-index, respectively. The vendors of the gas sensors recommend using an environmental sensor for measuring the temperature (T) and relative humidity (RH) of the environment. Thus, the SHCT3 environmental sensor (Sensirion AG, Stäfa, Switzerland) has been used for measuring the T and, RH and feed them to the SGP30, and SGP40 algorithm for calibrating the calculation of the IAQ-index and TVOC values. The calibration process required approximately 5 min before each time the sensor was turned on. All used sensors communicate with the WeMos D1 Mini microcontroller (MCU) using the I2C bus, which allows the MCU to communicate with all sensors at the same time using a unique I2C address for each sensor. After the calibration process is completed, the system starts to measure the TVOC and IAQ-index baseline at the system location. The system will measure both IAQ-index, and TVOC in parallel and in a normal clean environment. The system measurements should be kept in levels 1 and 2 for both gas sensors as one of the test conditions. Figure 6 explains the flowchart of the measurements process.

The system is designed to contentiously operate in the target environment. The measured parameters are sent to the IoT-cloud for monitoring and data storing. In the presented work, two test scenarios were used. The first test scenario was set up inside a typical hood (Waldner Holding GmbH and Co. KG, Wangen im Allgäu, Germany) designed for chemical and analytical laboratories. The sensor node was fixed on a movable stand with manual adjustable height. The testing VOCs were injected inside a petri dish using pipettes from Eppendorf (Eppendorf AG, Hamburg, Germany). The second test environment was an automated chemical laboratory (Center for Life Science Automation, University of Rostock, Germany) using a humanoid robot as a host for the sensor node.

## 4. Results

The aim of the tests in all scenarios and positions is to determine the minimum VOC amount that can be detected by the used sensors from the testing distance (distance between the VOC leakage source and the sensors). The system has been tested with 12 different VOC solvents, according to Table 2.

The tests were carried out at two chemical laboratories with different tests procedures. The first laboratory was a classical laboratory for manual sample preparation, whereas the second laboratory was used for automated sample preparation with stationary and humanoid robots. Both laboratories have an air conditioning system which stabilizes the laboratory temperature at approximately 22 ± 0.5 °C. Furthermore, both sensors have been provided by the temperature and humidity live measurements using the SHCT3 environmental sensor (included in the sensor node) to overcome any drift on the gas measurements due to unstable temperature and relative humidity. The following section describes the tests procedure and tests results in each test environment.

### 4.1. System Testing in Chemicals Preparation Hood

**Figure 7:**
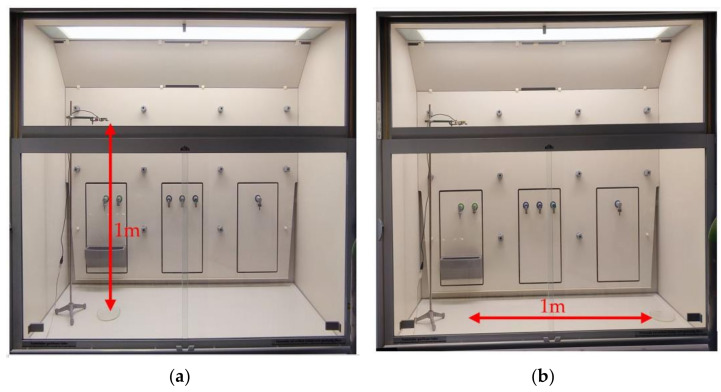
Testing positions for VOC source; (**a**)-position 1 m below the sensor, (**b**)-position 1 m away from the sensor.

These testes were carried out in a hood in a classical manual chemical laboratory. The hood has a special ventilation system for suction of chemical vapors during sample preparations. The ventilation system was turned off during the system testing to prevent any interference due to air ventilation. Two positions for the VOC source were used, one directly 1 m below the sensor. For the second test position, the sensor node was shifted one meter horizontally from the first position (see Figure 7).

### 4.2. H20 Tests

**Figure 8:**
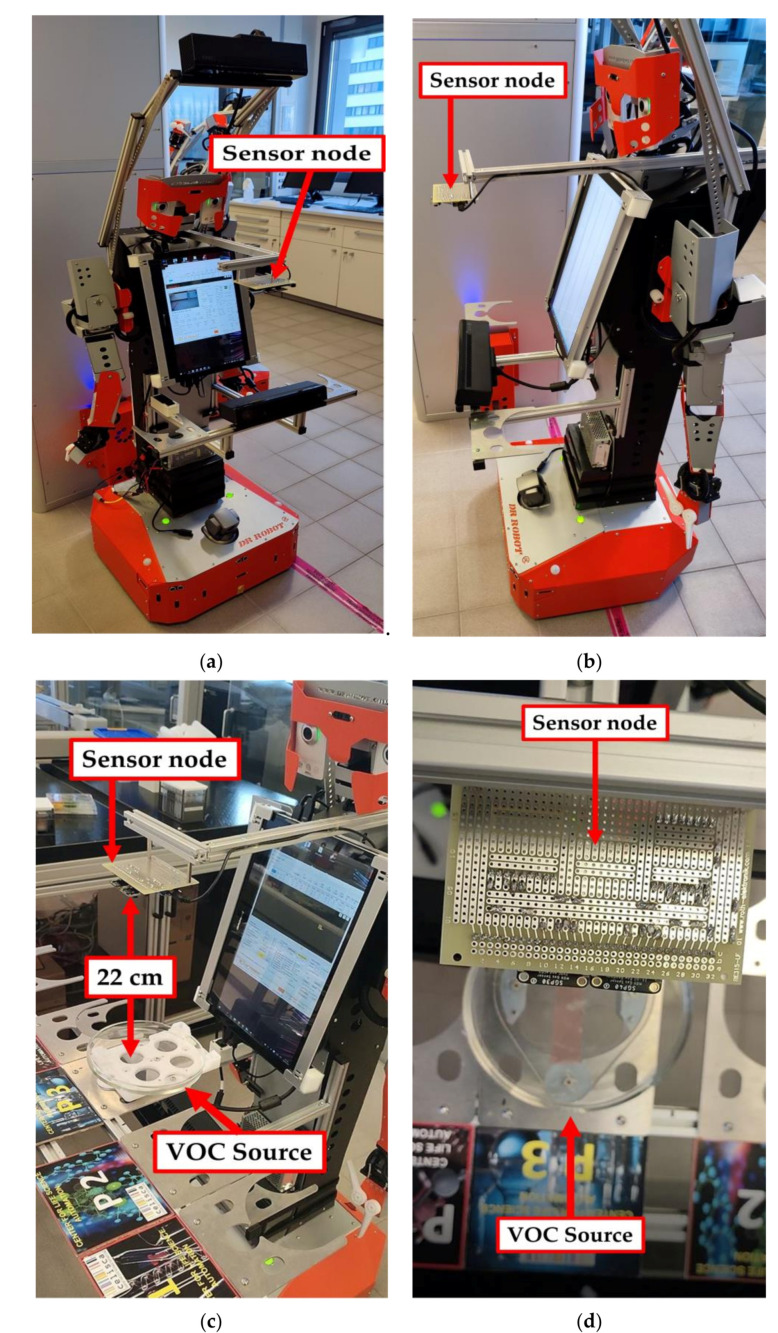
The sensor node hosted by the H20 robot. (**a**,**b**) front and side views of the used H20 robot with the sensor node. (**c**) The robot position for the measurements process. (**d**) The sensors are positioned directly above the VOCs leakage source for the testing process.

In the second testing scenario, the sensor node was mounted on an H20 humanoid robot (Dr. Robot Inc., Markham, ON, Canada). The H20 robot provides the required power supply voltage for operating the sensor node and for storing the acquired test results. The sensor node was fixed on the shoulder level of the robot body to allow the detection of any leakages of hazardous gases during sample preparation and transportation in automated chemical laboratories. In this scenario, the robot starts from a specific charging station and moves for one minute to reach the sample preparation table. The VOC material was injected inside the petri dish for the test. The robot was programmed to stop for 60 s directly at the leakage source (petri dish) which was located at 22 cm directly under the sensor node. After completion of the measurement time, the robot returns to the charging station. The total time from the starting until returning to the end point is approximately 3.5 min. Figure 8 shows the sensor node hosted by the H20 robot and the sensor node location regarding gas leakage source.

**Figure 9:**
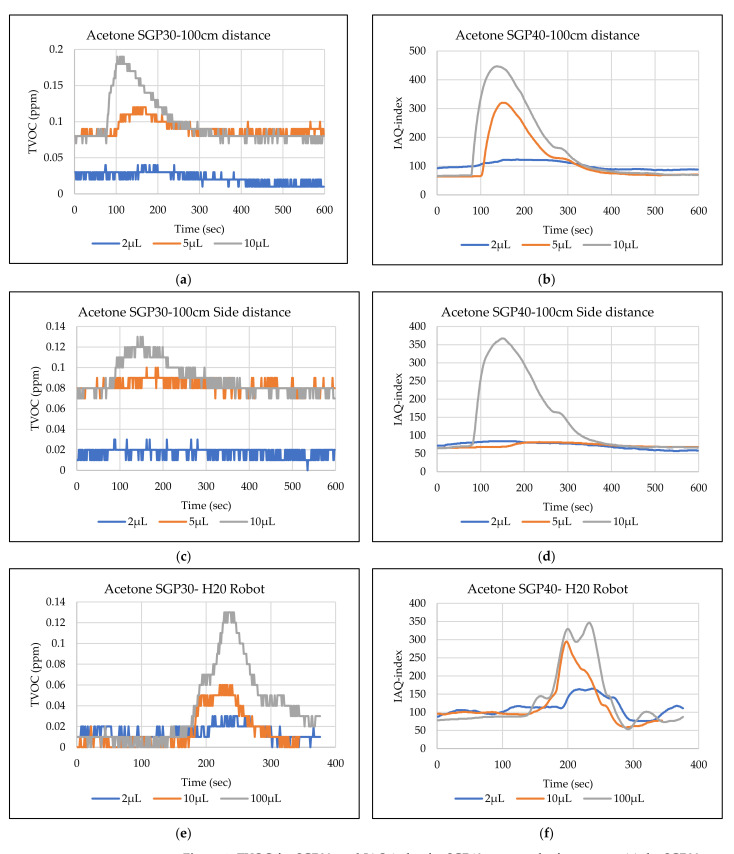
TVOC for SGP30, and IAQ-index for SGP40 tests results for acetone. (**a**) the SGP30 tests results from 1 m directly under the leakage source, (**b**) the SGP40 tests results from 1 m directly under the leakage source, (**c**) SGP30 tests results after shifting the leakage source 1 m to the side of the first position, (**d**) the SGP40 tests results after shifted the leakage source 1 m to the side of the first position, (**e**) the H20 robot-SGP30 tests results from 22 cm from the VOC leakage source, and (**f**) the H20 robot-SGP40 tests results from 22 cm from the VOC leakage source.

**Figure 10:**
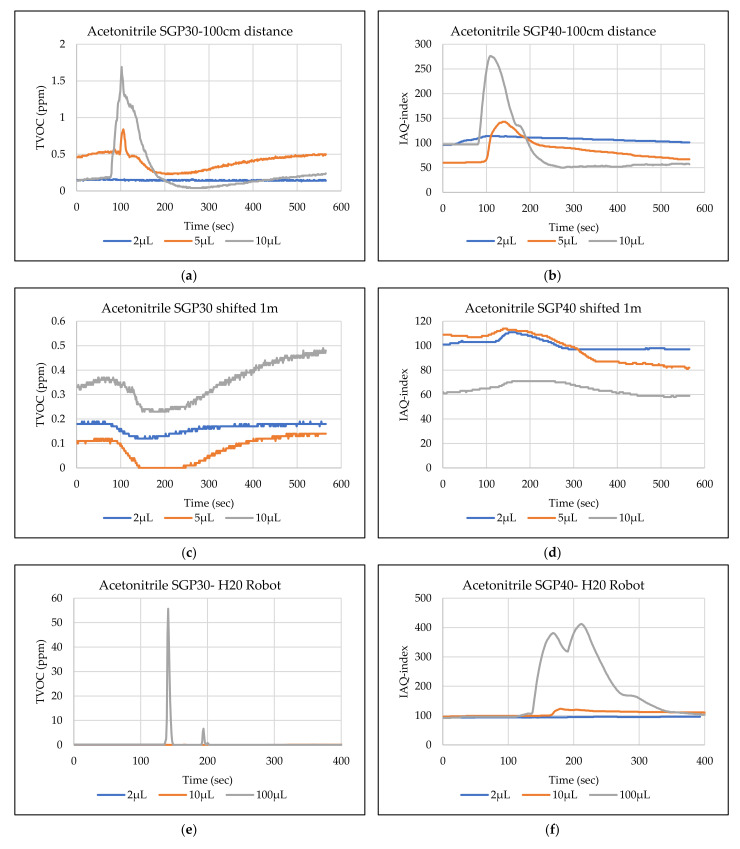
TVOC for SGP30, and IAQ-index for SGP40 tests results for acetonitrile. (**a**) the SGP30 tests results from 1 m directly under the leakage source, (**b**) the SGP40 tests results from 1 m directly under the leakage source, (**c**) SGP30 tests results after shifting the leakage source 1 m to the side of the first position, (**d**) the SGP40 tests results after shifted the leakage source 1 m to the side of the first position, (**e**) the H20 robot-SGP30 tests results from 22 cm from the VOC leakage source, and (**f**) the H20 robot-SGP40 tests results from 22 cm from the VOC leakage source.

**Figure 11:**
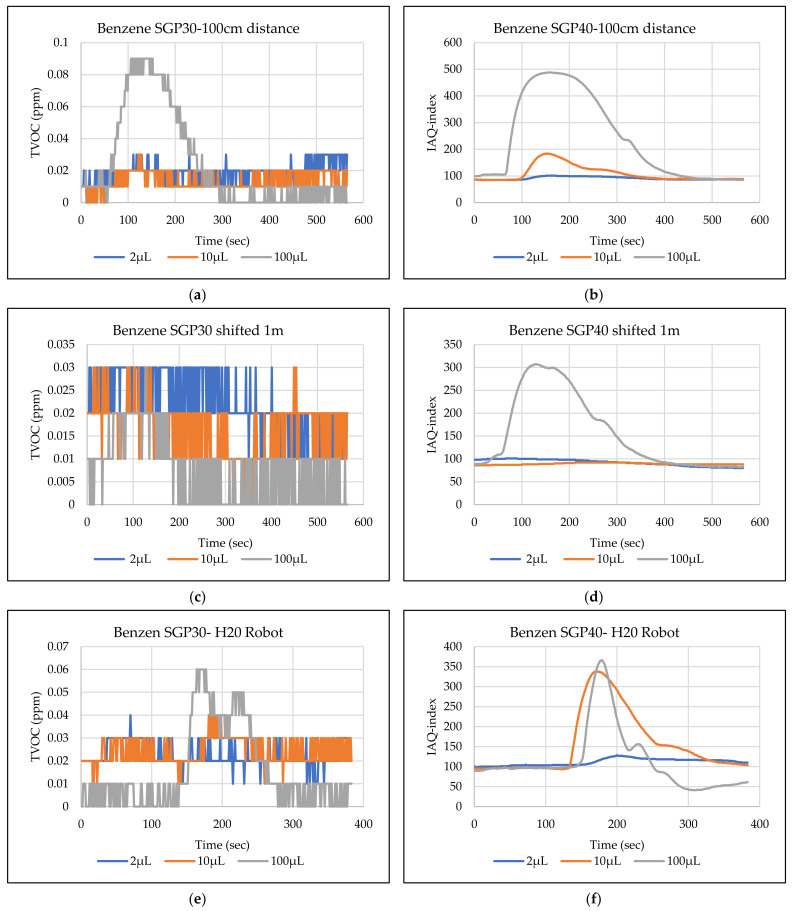
TVOC for SGP30, and IAQ-index for SGP40 tests results for benzene. (**a**) the SGP30 tests results from 1 m directly under the leakage source, (**b**) the SGP40 tests results from 1 m directly under the leakage source, (**c**) SGP30 tests results after shifting the leakage source 1 m to the side of the first position, (**d**) the SGP40 tests results after shifted the leakage source 1 m to the side of the first position, (**e**) the H20 robot-SGP30 tests results from 22 cm from the VOC leakage source, and (**f**) the H20 robot-SGP40 tests results from 22 cm from the VOC leakage source.

**Figure 12:**
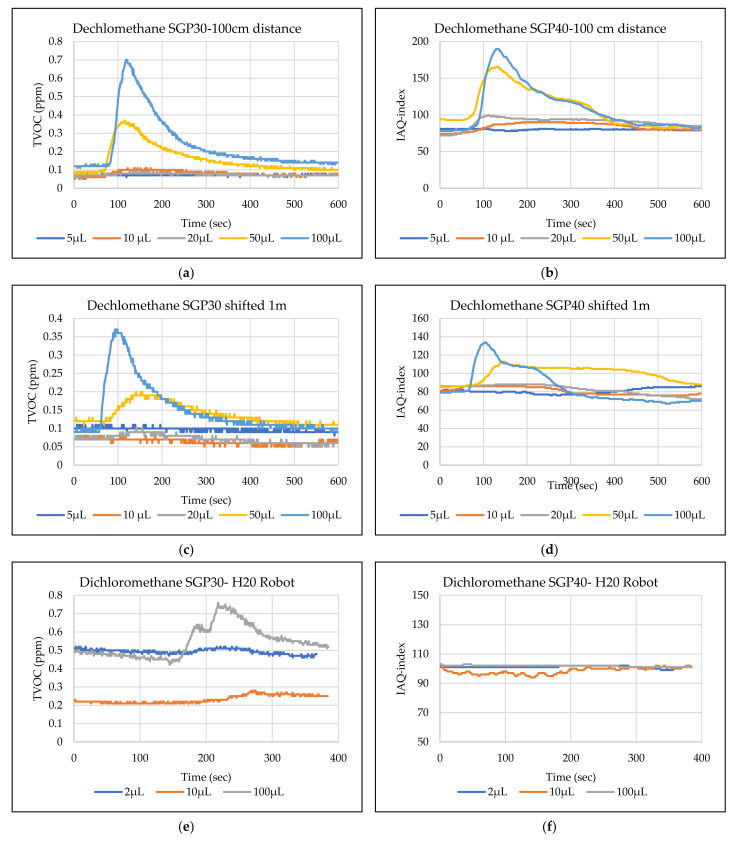
TVOC for SGP30, and IAQ-index for SGP40 tests results for Dichloromethane. (**a**) the SGP30 tests results from 1 m directly under the leakage source, (**b**) the SGP40 tests results from 1 m directly under the leakage source, (**c**) SGP30 tests results after shifting the leakage source 1 m to the side of the first position, (**d**) the SGP40 tests results after shifted the leakage source 1 m to the side of the first position, (**e**) the H20 robot-SGP30 tests results from 22 cm from the VOC leakage source, and (**f**) the H20 robot-SGP40 tests results from 22 cm from the VOC leakage source.

**Figure 13:**
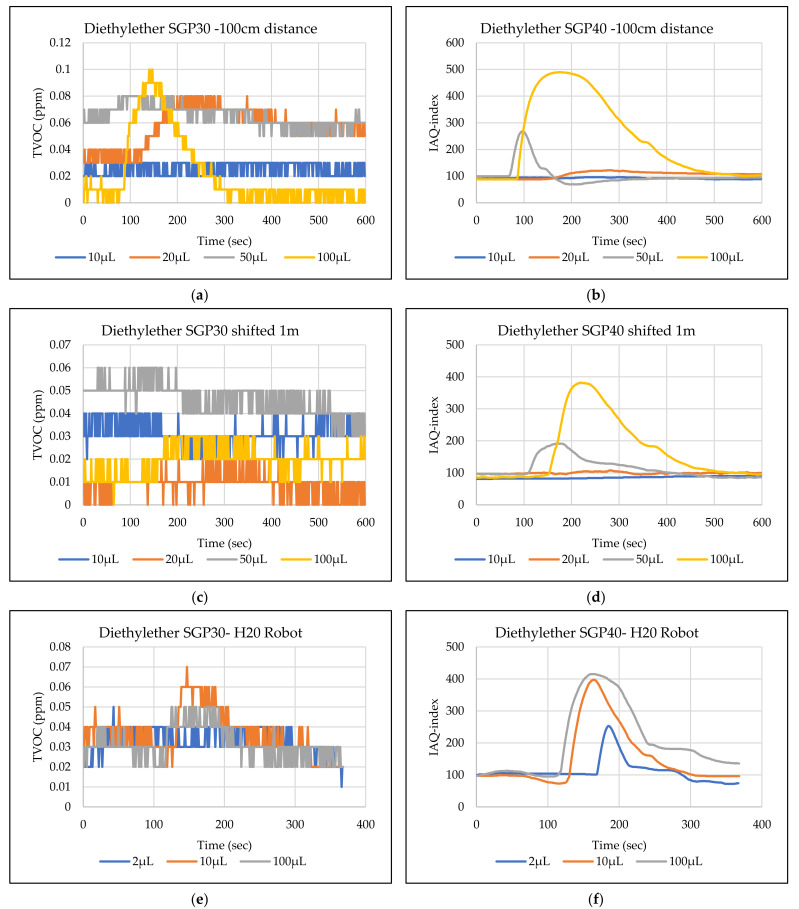
TVOC for SGP30, and IAQ-index for SGP40 tests results for Diethyl ether. (**a**) the SGP30 tests results from 1 m directly under the leakage source, (**b**) the SGP40 tests results from 1 m directly under the leakage source, (**c**) SGP30 tests results after shifting the leakage source 1 m to the side of the first position, (**d**) the SGP40 tests results after shifted the leakage source 1 m to the side of the first position, (**e**) the H20 robot-SGP30 tests results from 22 cm from the VOC leakage source, and (**f**) the H20 robot-SGP40 tests results from 22 cm from the VOC leakage source.

**Figure 14:**
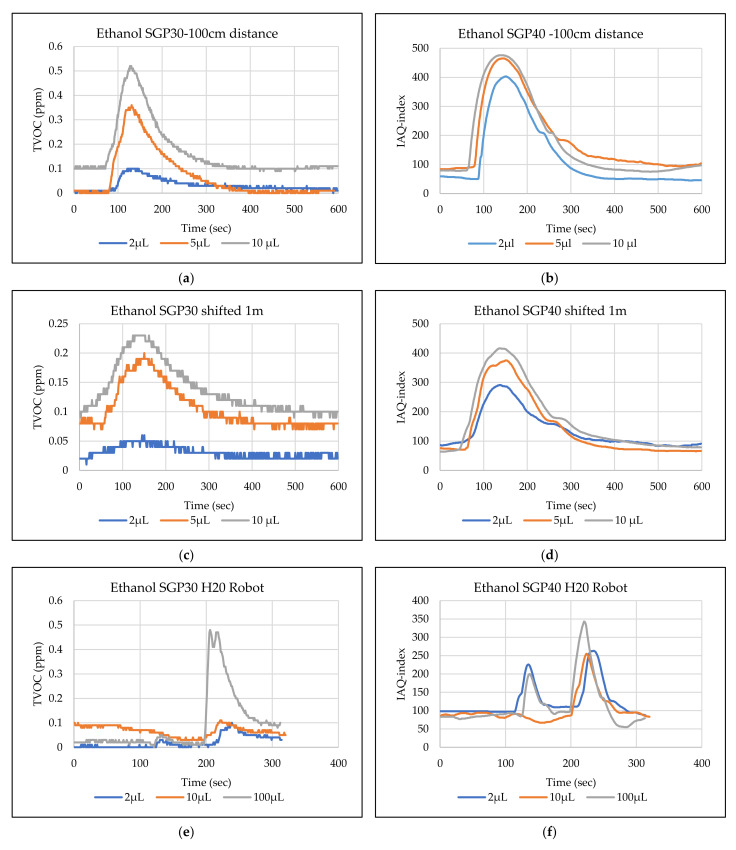
TVOC for SGP30, and IAQ-index for SGP40 tests results for ethanol. (**a**) the SGP30 tests results from 1 m directly under the leakage source, (**b**) the SGP40 tests results from 1 m directly under the leakage source, (**c**) SGP30 tests results after shifting the leakage source 1 m to the side of the first position, (**d**) the SGP40 tests results after shifted the leakage source 1 m to the side of the first position, (**e**) the H20 robot-SGP30 tests results from 22 cm from the VOC leakage source, and (**f**) the H20 robot-SGP40 tests results from 22 cm from the VOC leakage source.

**Figure 15:**
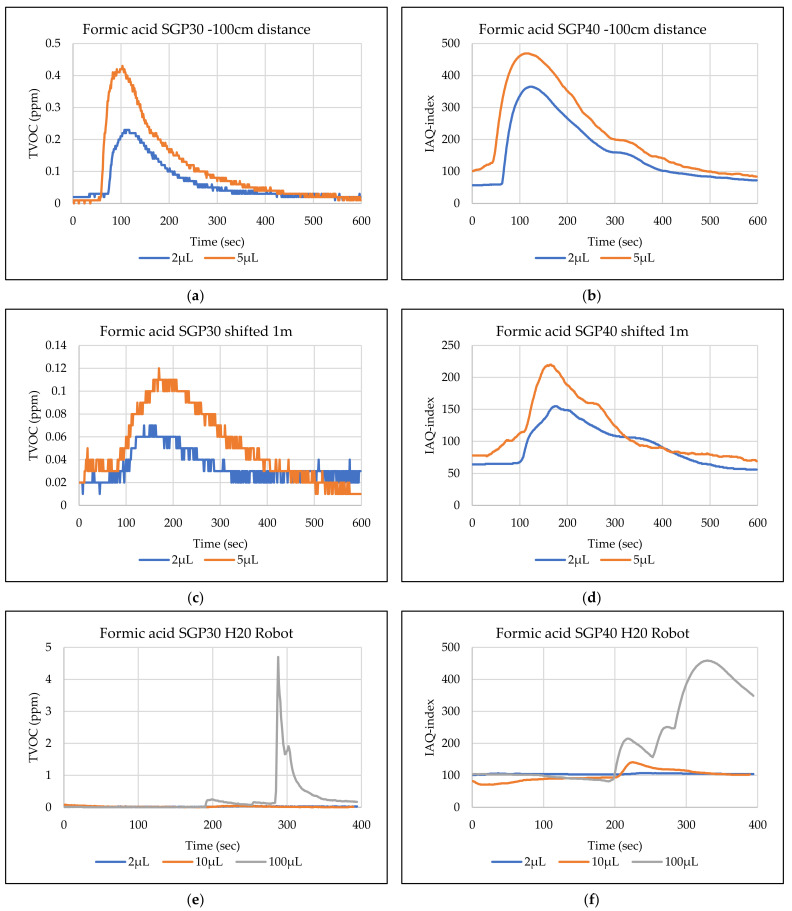
TVOC for SGP30, and IAQ-index for SGP40 tests results for formic acid. (**a**) the SGP30 tests results from 1 m directly under the leakage source, (**b**) the SGP40 tests results from 1 m directly under the leakage source, (**c**) SGP30 tests results after shifting the leakage source 1 m to the side of the first position, (**d**) the SGP40 tests results after shifted the leakage source 1 m to the side of the first position, (**e**) the H20 robot-SGP30 tests results from 22 cm from the VOC leakage source, and (**f**) the H20 robot-SGP40 tests results from 22 cm from the VOC leakage source.

**Figure 16:**
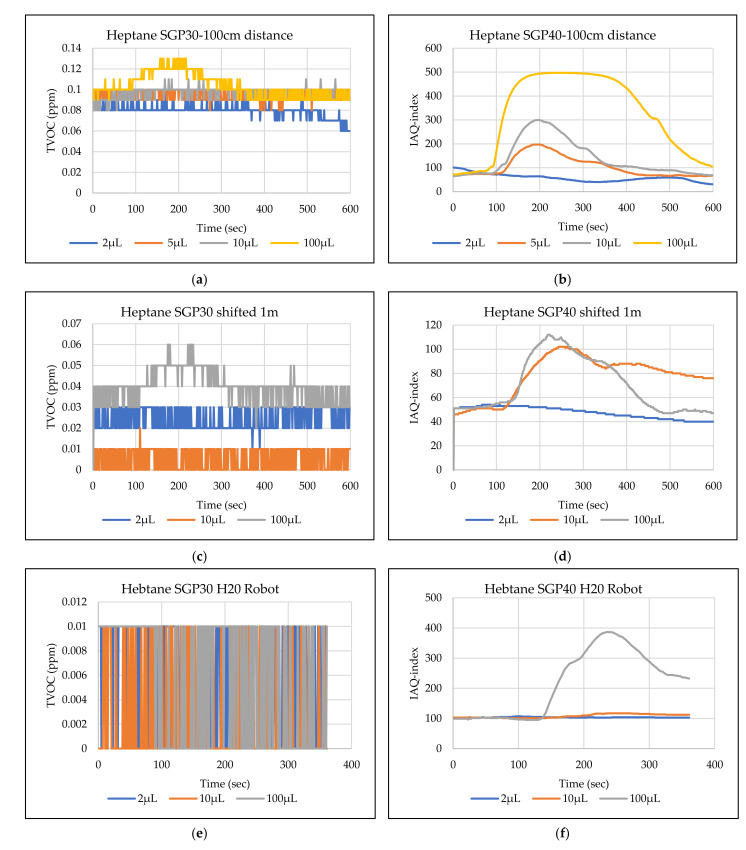
TVOC for SGP30, and IAQ-index for SGP40 tests results for heptane. (**a**) the SGP30 tests results from 1 m directly under the leakage source, (**b**) the SGP40 tests results from 1 m directly under the leakage source, (**c**) SGP30 tests results after shifting the leakage source 1 m to the side of the first position, (**d**) the SGP40 tests results after shifted the leakage source 1 m to the side of the first position, (**e**) the H20 robot-SGP30 tests results from 22 cm from the VOC leakage source, and (**f**) the H20 robot-SGP40 tests results from 22 cm from the VOC leakage source.

**Figure 17:**
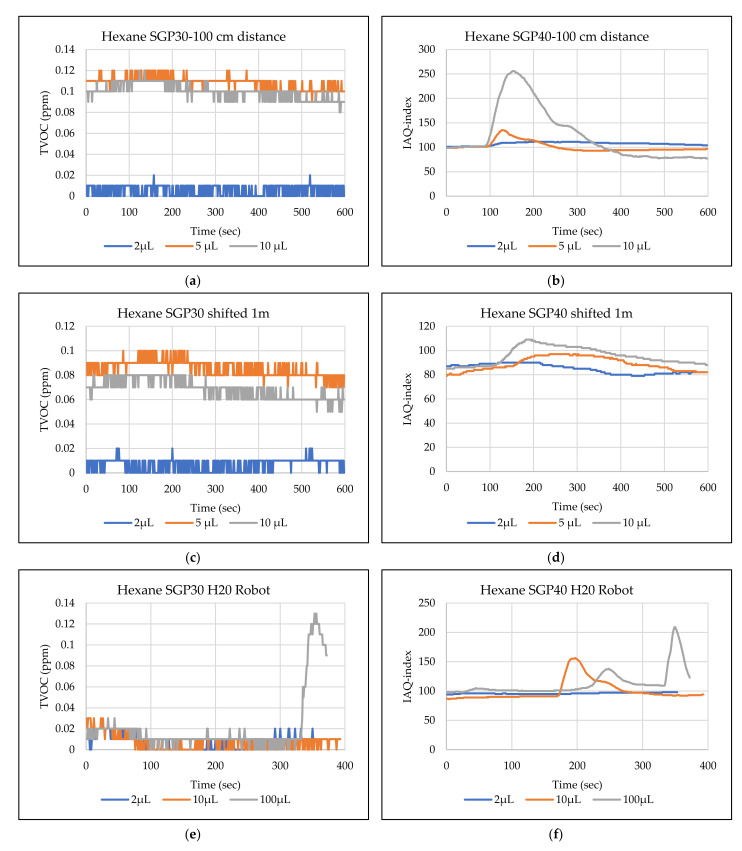
TVOC for SGP30, and IAQ-index for SGP40 tests results for hexane. (**a**) the SGP30 tests results from 1 m directly under the leakage source, (**b**) the SGP40 tests results from 1 m directly under the leakage source, (**c**) SGP30 tests results after shifting the leakage source 1 m to the side of the first position, (**d**) the SGP40 tests results after shifted the leakage source 1 m to the side of the first position, (**e**) the H20 robot-SGP30 tests results from 22 cm from the VOC leakage source, and (**f**) the H20 robot-SGP40 tests results from 22 cm from the VOC leakage source.

**Figure 18:**
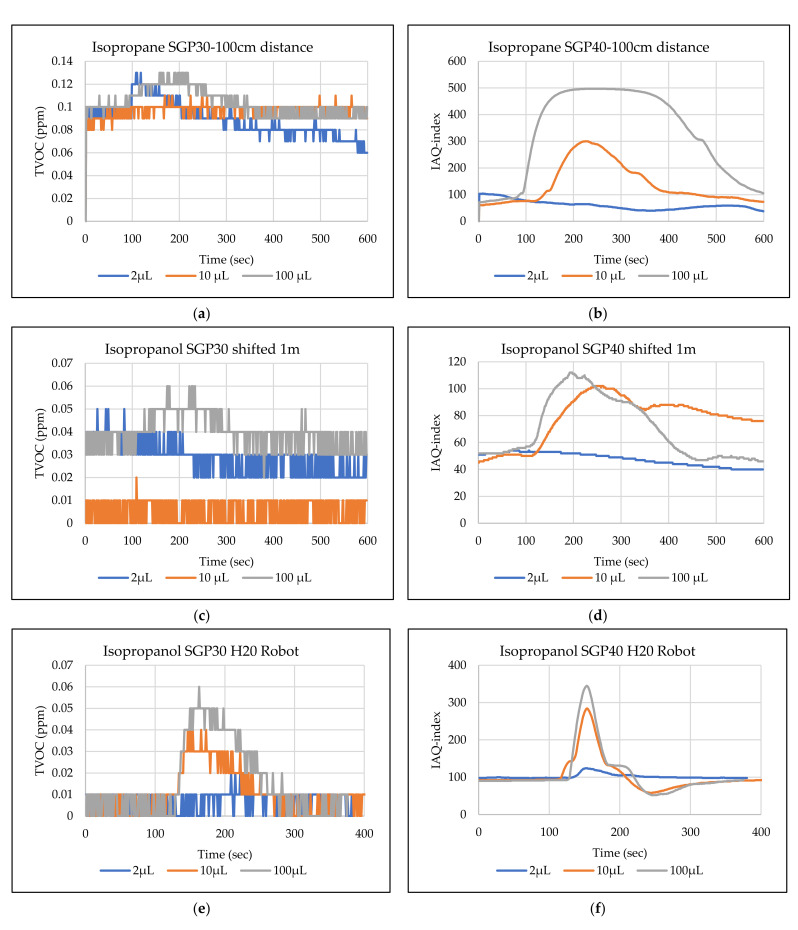
TVOC for SGP30, and IAQ-index for SGP40 tests results for isopropanol. (**a**) the SGP30 tests results from 1 m directly under the leakage source, (**b**) the SGP40 tests results from 1 m directly under the leakage source, (**c**) SGP30 tests results after shifting the leakage source 1 m to the side of the first position, (**d**) the SGP40 tests results after shifted the leakage source 1 m to the side of the first position, (**e**) the H20 robot-SGP30 tests results from 22 cm from the VOC leakage source, and (**f**) the H20 robot-SGP40 tests results from 22 cm from the VOC leakage source.

**Figure 19:**
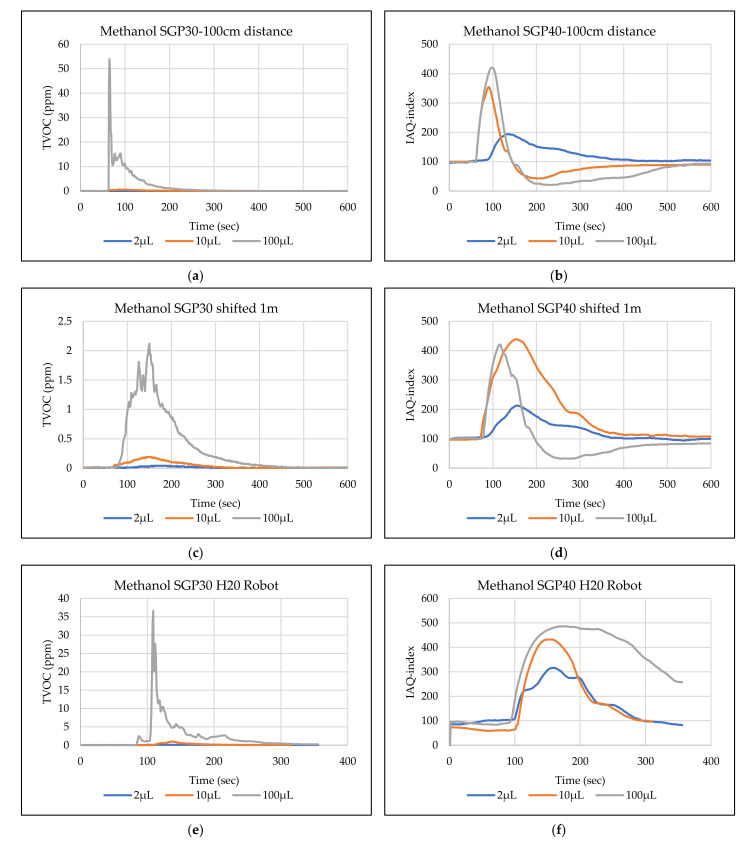
TVOC for SGP30, and IAQ-index for SGP40 tests results for methanol. (**a**) the SGP30 tests results from 1 m directly under the leakage source, (**b**) the SGP40 tests results from 1 m directly under the leakage source, (**c**) SGP30 tests results after shifting the leakage source 1 m to the side of the first position, (**d**) the SGP40 tests results after shifted the leakage source 1 m to the side of the first position, (**e**) the H20 robot-SGP30 tests results from 22 cm from the VOC leakage source, and (**f**) the H20 robot-SGP40 tests results from 22 cm from the VOC leakage source.

**Figure 20:**
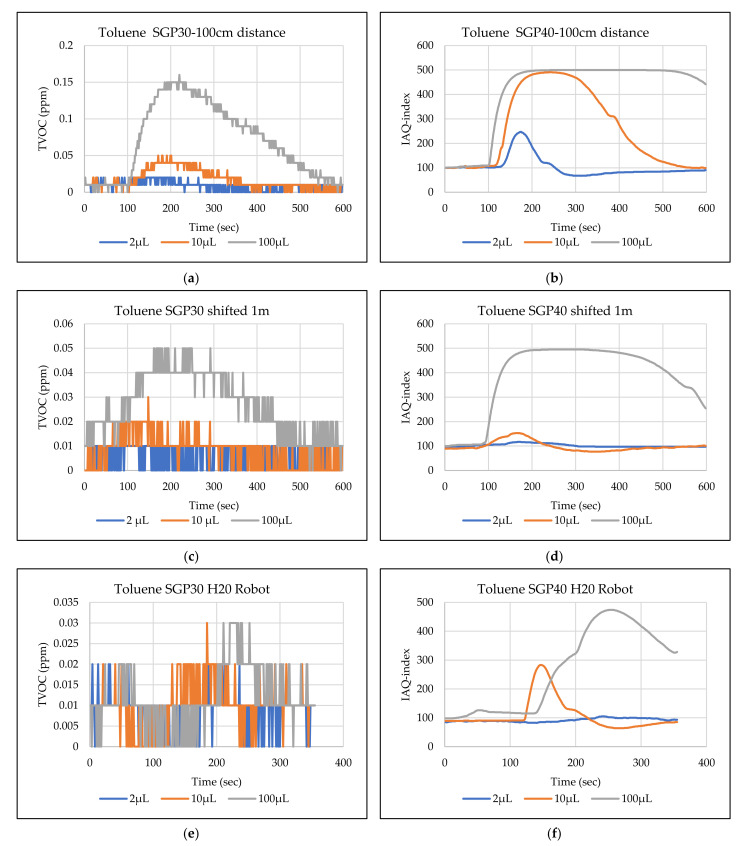
TVOC for SGP30, and IAQ-index for SGP40 tests results for toluene. (**a**) the SGP30 tests results from 1 m directly under the leakage source, (**b**) the SGP40 tests results from 1 m directly under the leakage source, (**c**) SGP30 tests results after shifting the leakage source 1 m to the side of the first position, (**d**) the SGP40 tests results after shifted the leakage source 1 m to the side of the first position, (**e**) the H20 robot-SGP30 tests results from 22 cm from the VOC leakage source, and (**f**) the H20 robot-SGP40 tests results from 22 cm from the VOC leakage source.

The main goal of the tests is the determination of the minimum volume of leakages (VOCs) that can be detected by the sensor node. Thus, the tests have been carried out with different volumes. Depending on the sensor’s response, the volume was increased. The used volumes were 2 µL, 5 µL, 10 µL, 50 µL, and 100 µL. Once the minimum detectable volume for a selected position and distance was determined, the test volume was not increased any further in order to avoid overloading the sensor. Figure 9, Figure 10, Figure 11, Figure 12, Figure 13, Figure 14, Figure 15, Figure 16, Figure 17, Figure 18, Figure 19 and Figure 20 below show the test results of the selected VOCs inside the hood for two locations of the petri dish and for sensor node with H20 robot. Each figure has 6 charts (a–f) presenting (a) the SGP30 tests results from 1 m directly under the leakage source, (b) the SGP40 tests results from 1 m directly under the leakage source, (c) SGP30 tests results after shifting the leakage source 1 m to the side of the first position, (d) the SGP40 tests results after shifted the leakage source 1 m to the side of the first position, (e) the H20 robot-SGP30 tests results from 22 cm from the VOC leakage source, and (f) the H20 robot-SGP40 tests results from 22 cm from the VOC leakage source.

**Figure 21:**
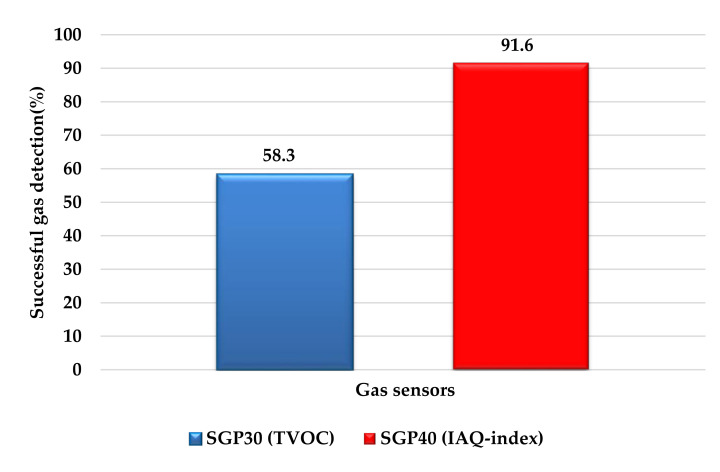
Percentage of successful gas detection attempts for both sensors.

In the experimental testing, each gas sensor was tested 36 times with all the 12 tested VOCs. The SGP40 successfully detects the tested VOCs 33/36 while the SGP30 sensor success in only 21/36 attempts. Figure 21 summarized the percentage of successful gas detection attempts for both sensors.

## 5. Discussion

The SGP30 and SGP40 tests result give us a clear view of individual sensor performance and the sensor detection range for each solvent for two positions inside the hood, as well as attached with the humanoid H20 robot. The two sensors reacted differently for each tested VOC material, as shown in Figure 9, Figure 10, Figure 11, Figure 12, Figure 13, Figure 14, Figure 15, Figure 16, Figure 17, Figure 18, Figure 19 and Figure 20. We can summarize the acquired result for each tested material as follows:

- Acetone: the tests show that the TVOC SGP30 sensor can detect volumes ≥ 10 µL inside the hood for both positions (directly under the sensor and shifted one meter), while the IAQ-index of SGP40 can detect volumes ≥ 5 µL directly under the sensor and ≥10µL for shifted 1 m horizontally. The H20 robot tests show a weak sensor signal of the SGP30 sensor for 2 µL and 10 µL. A sufficient signal that can be used as a threshold for the alarm systems can only be achieved for volumes ≥ 100 µL. The H20 robot tests for SGP40 show better performance and the sensor can clearly react and detect volumes ≥ 10 µL.
- Acetonitrile: the tests show that the TVOC of the SGP30 sensor can detect volumes ≥ 10 µL inside the hood for the first position, and failed to detect the 2 µL, 5 µL, 10 µL for the second position. The IAQ-index of SGP40 can detect volumes ≥ 5µL directly under the sensor and failed to detect the 2 µL, 5 µL, 10 µL for the second position. The H20 robot tests show that the SGP30 can only detect the volume ≥ 100 µL. The H20 robot tests for SGP40 show better performance and the sensor can clearly react and detect volumes ≥ 10 µL.
- Benzene: the tests show that the TVOC of the SGP30 sensor can detect volumes ≥ 100 µL inside the hood for the first position but failed for the detection of volumes up to 100 µL for the second position. The IAQ-index of SGP40 can detect volumes ≥ 10 µL directly under the sensor, as well as volumes ≥ 100 µL for the second position. The H20 robot tests show only a weak signal for the SGP30 and reacts weakly for a volume of 100 µL. The H20 robot tests for the SGP40 show better performance; the sensor can clearly react and detect volumes ≥ 10 µL.
- Dichloromethane: the tests show that the TVOC of the SGP30 sensor can detect volumes ≥ 100 µL inside the hood for both positions. The IAQ-index of the SGP40 can detect the volumes ≥ 50 µL directly under the sensor and volumes ≥ 100 µL for the second position. In the H20 robot tests, the SGP30 can detect volumes ≥ 100 µL, whereas no detection was possible with the SGP40.
- Diethyl ether: the tests show a weak reaction of the TVOC of the SGP30 for all tested volumes at both positions. The IAQ-index of the SGP40 enables the detection of volumes ≥ 50 µL for both positions. The H20 robot tests again show a weak reaction of the SGP30 sensor for all tested volumes. The H20 robot tests for SGP40 show better performance and enable detection of volumes ≥ 2 µL.
- Ethanol: inside the hood, the TVOC of the SGP30 sensor can detect volumes ≥ 5 µL inside the hood for the first position. A slightly weaker response can be found for the second position. The IAQ-index of SGP40 can detect volumes ≥2 µL for both positions. The H20 robot tests show that the SGP30 can detect volumes ≥ 100 µL, whereas the SGP40 shows better performance and enables the detection of volumes ≥ 2 µL.
- Formic acid: the TVOC of the SGP30 sensor enables the detection of volumes ≥ 2 µL inside the hood for the first position, and ≥5 µL for the second position, respectively. The IAQ-index of SGP40 can detect volumes ≥ 2 µL for both positions. The H20 robot tests show that the SGP30 and SGP40 can detect volumes ≥ 100 µL.
- Heptane: for all tested volumes, no sensor signals were detected for the TVOC of the SGP30 in both positions. The IAQ-index of SGP40 can detect volumes ≥ 5 µL for both positions. The H20 robot tests again show that the SGP30 cannot detect volumes below 100 µL. In contrast, the tests for the SGP40 resulted in minimum detectable volumes ≥ 100 µL.
- Hexane: the tests show that the TVOC of the SGP30 sensor cannot detect the tested volumes for both positions. The IAQ-index of SGP40 can detect volumes ≥ 5 µL for the first position but failed for the second position. The H20 robot tests show that the SGP30 can detect volumes ≥ 100 µL, whereas the SGP40 enables the detection of volumes ≥ 10 µL.
- Isopropanol: the tests show a weak response that the TVOC of the SGP30 sensor reacts weak for all the 2 µL, 10 µL, and 100 µL for both positions. The IAQ-index of SGP40 can detect the volumes ≥ 10 µL for both positions. The H20 robot tests show that the SGP30 reacts weak for all tested volumes, whereas the SGP40 shows a better performance and can clearly detect volumes ≥ 10 µL.
- Methanol: the tests inside the hood show that the TVOC of the SGP30 sensor can detect volumes ≥ 100 µL for both positions. The IAQ-index of the SGP40 showed a good signal for volumes ≥ 2 µL for both positions. In the H20 robot tests, volumes ≥ 100 µL can be detected with the SGP 30. The SGP40 again shows better performance and enables the detection of volumes ≥ 2 µL.
- Toluene: the tests inside the hood show that the TVOC of the SGP30 sensor can detect volumes ≥ 100 µL for the first position, whereas only a weak signal could be found for all tested volumes for the second position. The IAQ-index of the SGP40 can detect volumes ≥ 2 µL directly under the sensor and volumes ≥ 100 µL for the second position. In the H20 robot tests, no signals were detected for all tested volumes for the SGP30. The SGP40 enables the detection of volumes ≥ 10 µL.

Table 3 explain the SGP30 and SGP40 gas sensors tests result for the minimum detected useful volumes of the tested VOC solvents in all test environments and positions where P1 refer to the test inside the hood directly one meter under the sensor node, and P2 refer to the same test inside the hood with shafting the leakage source one meter.

**Figure 22:**
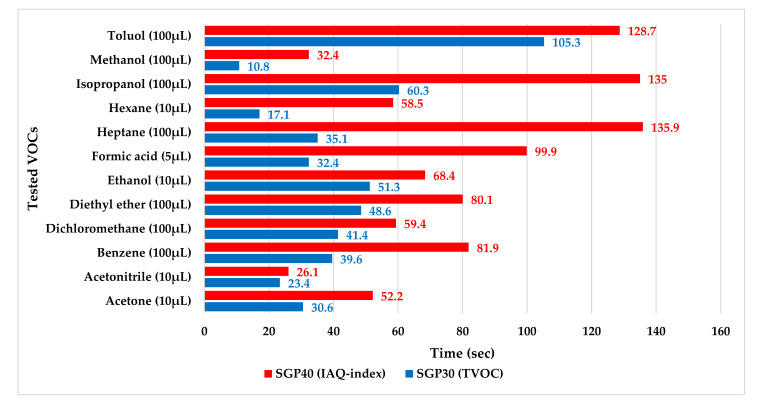
Response time for SGP30 and SGP40 gas sensors for several tested VOCs.

The sensor’s response time (the time required for the sensor output signal to reach 90% of the maximum measured value from its previous settled state) for each tested VOC material was calculated for position 1 (inside the hood directly 1 m under the tested material). Figure 22 shows the sensor response time for both SGP30 and SGP40 gas sensors.

## 6. Conclusions

In this work, we investigate the performance of two novel gas sensors SPG30, and SPG40 that use different parameters for detecting hazardous and toxic gases/chemical vapors which are the TVOC, and IAQ-index. One of the main goals of this study is to help us to select the best parameter between TVOC and IAQ-index for future design and implementation of hazardous gases detection and alarming systems. The tests were implemented in two procedures in different test environments the first inside a chemical preparation hood and the second in an open automated laboratory by attaching the sensor node to a Mobil robot. The tests result show that the IAQ-index of the SGP40 reacts better compared to the TVOC of SGP30 for 11 of 12 tested VOCs materials (except the dichloromethane). The SGP40 sensor showed better test results and successfully detects the gas leakage in 91.6% of the test attempts while the SGP30 was only successful in 58.3% of the test attempts. The sensor response time for all tested VOCs material was calculated for both sensors, and the results show that the response time of the SGP40 sensor is longer for all the tested VOCs. Based on these results, the TVOC based SGP30 gas sensor will be replaced with IAQ-index based SGP40 or a newer version that uses the IAQ-index for air quality and gas impurity levels detection for new sensor nodes.

## Figures and Tables

**Table 1:** IAQ-index and TVOC categories concerning the IAQ levels

IAQ Levels	Category	IAQ-Index Values	TVOC [ppm]
1	Good	0–50	0–0.065
2	Moderate	51–100	0.066–0.22
3	Unhealthy for sensitive peoples	101–150	0.23–0.66
4	Unhealthy	151–200	0.67–2.2
5	Very unhealthy	201–300	2.3–5.5
6	Hazardous	301–500	>5.5

**Table 2:** The Tested VOC solvents.

Name	Molecular Formula	Boiling Point in °C
Acetone	C_3_H_6_O	56
Acetonitrile	C_2_H_3_N	82
Benzene	C_6_H_6_	80.1
Dichloromethane	CH_2_Cl_2_	39.6
Diethyl ether	C4H10O	34.6
Ethanol	C_2_H_6_O	78.37
Formic acid	CH_2_O_2_	100.8
Heptane	C_7_H_16_	98.42
Hexane	C_6_H_14_	69
Isopropanol	C_3_H_8_O	82.5
Methanol	CH_3_OH	64.7
Toluene	C_7_H_8_	110.6

**Table 3:** System Tests Results inside the Hood and when Attached to the H20 Robot.

VOC	SGP30-P1	SGP30-P2	SGP30-H20	SGP40-P1	SGP40-P2	SGP40-H20
Acetone	≥10 μL	≥10 μL	≥100 μL	≥5 μL	≥10 μL	≥10 μL
Acetonitrile	≥10 μL	-	≥100 μL	≥5 μL	-	≥10 μL
Benzene	≥100 μL	-	-	≥10 μL	≥100 μL	≥10 μL
Dichloromethane	≥100 μL	≥100 μL	≥100 μL	≥50 μL	≥100 μL	-
Diethyl ether	-	-	-	≥50 μL	≥50 μL	≥2 μL
Ethanol	≥5 μL	≥10 μL	≥100 μL	≥2 μL	≥2 μL	≥2 μL
Formic acid	≥2 μL	≥5 μL	≥100 μL	≥2 μL	≥2 μL	≥100 μL
Heptane	-	-	≥100 μL	≥5 μL	≥5 μL	≥100 μL
Hexane	-	-	≥100 μL	≥5 μL	-	≥10 μL
Isopropanol	-	-	-	≥10 μL	≥10 μL	≥10 μL
Methanol	≥100 μL	≥100 μL	≥100 μL	≥2 μL	≥2 μL	≥2 μL
Toluene	≥100 μL	-	-	≥2 μL	≥100 μL	≥10 μL

## Data Availability

The data presented in this work are available on request from the corresponding author.
